# Semantic fluency deficits and associated brain activity in Parkinson’s disease with mild cognitive impairment

**DOI:** 10.1007/s11682-022-00698-7

**Published:** 2022-07-16

**Authors:** Jihyun Yang, Katie L McMahon, David A Copland, Dana Pourzinal, Gerard J Byrne, Anthony J Angwin, John D O’Sullivan, Nadeeka N Dissanayaka

**Affiliations:** 1 UQ Centre for Clinical Research, Faculty of Medicine, The University of Queensland, Herston, Queensland Australia; 2 School of Clinical Sciences and Centre for Biomedical Technologies, Queensland University of Technology, Brisbane, Queensland Australia; 3 School of Health & Rehabilitation Sciences, The University of Queensland, St Lucia, Queensland Australia; 4 Mental Health Service, Royal Brisbane & Women’s Hospital, Herston, Queensland Australia; 5 Department of Neurology, Royal Brisbane & Women’s Hospital, Herston Queensland, Australia; 6 School of Psychology, The University of Queensland, St Lucia, Queensland Australia

**Keywords:** Parkinson’s disease, Mild cognitive impairment, Functional magnetic resonance imaging, Semantic fluency

## Abstract

People living with Parkinson’s disease (PD) with poor verbal fluency have an increased risk of developing dementia. This study examines the neural mechanisms underpinning semantic fluency deficits in patients with PD with mild cognitive impairment (PD-MCI) compared to those without MCI (PD-NC) and control participants without PD (non-PD). Thirty-seven (37) participants with PD completed a cognitive assessment battery to identify MCI (13 PD-MCI). Twenty sex- and age-matched non-PD patients also participated. Participants were scanned (3T Siemens PRISMA) while performing semantic fluency, semantic switching, and automatic speech tasks. The number of responses and fMRI data for semantic generation and semantic switching were analyzed. Participants also completed a series of verbal fluency tests outside the scanner, including letter fluency. Participants with PD-MCI performed significantly worse than PD-NC and non-PD participants during semantic fluency and semantic switching tasks. PD-MCI patients showed greater activity in the right angular gyrus than PD-NC and non-PD patients during semantic switching. Increased right angular activity correlated with worse verbal fluency performance outside the scanner. Our study showed that the PD-MCI group performed worse on semantic fluency than either the PD-NC or non-PD groups. Increased right angular gyrus activity in participants with PD-MCI during semantic switching suggests early compensatory mechanisms, predicting the risk of future dementia in PD.

## Introduction

There is a growing interest in language impairment in Parkinson’s disease (PD). Notably, verbal fluency tasks have been widely used to further understand cognitive impairment in PD (Dujardin et al., 2004; Pagonabarraga & Kulisevsky, 2012; Rodriguez-Porcel et al., 2020). Verbal fluency tasks involve generating words that begin with a particular letter (letter fluency) or from a particular semantic category (semantic fluency) within a given period of time. Semantic fluency is strongly linked to semantic memory, whereas letter fluency relies less on semantic memory and more on executive functioning (Greenaway et al., 2009). Semantic switching, which requires switching between two semantic categories, is linked to frontal-executive control for alternating attention and inhibiting irrelevant responses (Ellfolk et al., 2014). Not only are PD patients with semantic fluency deficits more susceptible to dementia than those without semantic fluency deficits (Janvin et al., 2006; Williams-Gray et al., 2007, 2009), but they also report poorer quality of life and increased carer burden (Rosenthal et al., 2017). Given the clinical significance of semantic fluency impairment in PD, the present study investigated the neural underpinnings of semantic fluency deficits in PD with early cognitive deficits (i.e., mild cognitive impairment (MCI)) to explore a potential functional neural marker prior to the development of dementia.

Semantic fluency is associated with verbal ability and the anterior region of the left middle frontal gyrus and posterior regions of the temporal cortex, predominantly in the left fusiform gyrus (Baldo et al., 2006; Birn et al., 2010; Costafreda et al., 2006). A recent meta-analysis also reported activation of the left middle frontal gyrus, anterior cingulate gyrus, left parietal precuneus and subcortical areas, including the insula, thalamus, putamen, caudate, and claustrum, during both phonemic and semantic fluency in healthy adults (Wagner et al., 2014). The involvement of the temporal lobe in semantic fluency may reflect its role in the storage of semantic memories, while the prefrontal cortex plays a role in the executive control of semantic processing, such as the retrieval and control of information (Noppeney et al., 2004). Therefore, semantic fluency impairment in PD can be attributed to deficits in semantic memory (Guidi et al., 2015), cognitive set-shifting (Henry & Crawford, 2004), and additional retrieval mechanisms (Obeso et al., 2012). Although it is suggested that posterior cortical and temporal lobe dysfunction are associated with semantic fluency deficits in PD (Williams-Gray et al., 2009), to date, there are no studies investigating specific brain regions constituting this link.

The present study is novel in examining deficits in semantic fluency and associated neural activity in PD-MCI compared to PD without MCI (PD-NC) and control participants without PD (non-PD) using an overt semantic and semantic switching fluency fMRI task. It is predicted that a greater impairment in semantic and semantic switching fluency will be observed in participants with PD-MCI than in other groups and that impaired posterior cortical and temporal lobe activity will be related to semantic fluency deficits in PD-MCI.

### Methods

#### Participants

Thirty-seven (37) nondemented participants with PD and 20 age- and gender-matched non-PD controls were included in this study (Table 1). The inclusion criterion for PD was the fulfilment of the UK PD Society Brain Bank diagnostic criteria. Participants were included if their first language was English. Exclusion criteria were (1) not having English as their first language and the presence of (2) dementia diagnosed by their neurologist or possible dementia if the participants scored < 19 in the Montreal Cognitive Assessment (MoCA)(Nasreddine et al., 2005); (3) other neurological diseases; (4) major depression and/or significant anxiety screened by Mini International Neuropsychiatric Interview (MINI-plus); (5) contraindications for MRI; (6) clinically significant abnormal brain finding (e.g., cerebrovascular disease, meningioma) assessed by a neuroradiologist; or (7) excessive head movement > 1 voxel (2.6 mm^3^). All assessments in PD participants were performed in their ‘ON’ state (two PD participants were not on any PD medication). Written informed consent was collected from each participant prior to their participation. This study was approved by the human research ethics committees of the University of Queensland and the Royal Brisbane and Women’s Hospital.

**Table 1:** Demographics and clinical characteristics of participants

	PD-MCI (N = 13)	PD-NC (N = 24)	Non-PD (N = 20)	p-value	Post-hoc analysis
Sex (M/F)^a^	8/4	13/11	12/9	ns	
Age (years)^c^	70.38 (8.61, 54–83)	67.75 (9.29, 52–83)	64.00(8.08, 50–77)	ns	
Education (years)^b^	12.31 (3.54, 6–16)	13.22 (3.67, 6–18)	14.66(3.36, 10–20)	ns	
Disease Duration (Year)^d^	6.00 (3.70, 0–13)	4.29 (3.38, 0–12)		ns	
HY Stage^e^	1.62 (0.65, 1–3)	1.50 (0.66, 1–3)		ns	
LEDD (mg)^d^	368.27 (220.36, 0-713)	509.7 3(273.38, 0-1056)		ns	
MDS-UPDRS Total^e^	34.85 (12.05, 19–61)	32.96 (15.01, 14–75)		ns	
UPDRS-III^e^	20.46 (8.24, 8–33)	14.92 (9.12, 3–36)		ns	
MoCA^c^	23.00 (2.24, 20–27)	26.08 (2.21, 21–30)	26.52 (1.83, 22–29)	0.0001	PD-MCI < PD-NC***, < Non-PD***
CCRT ^b^	37.08 (8.46, 19–47)	38.08 (6.16, 26–48)	40.57 (6.87, 18–49)	ns	
HAM-A ^b^	4.23 (3.72, 0–10)	5.00 (4.18, 0–15)	0.94 (1.34, 0–5)	0.003	Non-PD < PD-MCI*, < PD-NC**
PAS^e^	7.46 (5.47, 2–19)	8.00 (7.05, 0–29)		ns	
HAM-D ^b^	4.38 (3.69, 0–12)	4.58 (3.34, 0–14)	1.94 (2.16, 0–8)	0.014	Non-PD < PD-NC*
SAS ^b^	11.31 (4.99, 3–18)	10.09 (5.15, 1–22)	9.40 (3.58, 3–16)	ns	
IADL^e^	M 7.88 (0.33, 7–8) F 7.67 (0.58, 7–8)	M 7.50 (0.79, 6–8) F 7.73 (0.65, 6–8)		ns	
IQCODE^e^	3.01(0.45. 2–3)	3.28(0.33, 3–4)			
DKEFS Letter^b^	24.08 (0.87, 10–43)	40.79 (10.42, 23–62)	46.90 (16.00, 25–84)	0.001	PD-MCI < PD-NC***, < Non-PD***
DKEFS Semantic^b^	29.23 (7.36, 17–45)	38.92 (9.37, 25–65)	43.55 (12.00, 25–77)	0.001	PD-MCI < PD-NC**, < Non-PD***
DKEFS Semantic Switching^c^	11.23 (2.09, 9–15)	13.50 (3.05, 10–22)	14.90 (3.35, 10–22)	0.004	PD-MCI < Non-PD**

^a^ Chi-square Test; ^b^Kruskal-Wallis H Test; ^c^ANOVA (Analysis of Variance) Test; ^d^ Independent T-Test; ^e^ Mann Whitney Test Results are presented as mean (SD, range). Statistically significant results for post-hoc analysis are indicated as * *p* < 0.05, ** *p* < 0.001, *** *p* < 0.0001. *Note*. PD-MCI = Parkinson’s disease participants with mild cognitive impairment; PD-NC = Parkinson’s disease participants with no cognitive impairment; non-PD = control participants without PD; HY Stage = Hoehn and Yahr Stage (1–5); LEDD = Levodopa Equivalent Daily Dose; MDS-UPDRS = Movement Disorder Society Unified Parkinson’s Disease Rating Scale; MoCA = Montreal Cognitive Assessment; PDCRS = Parkinson’s Disease Cognitive Rating Scale; CCRT = Cambridge Contextual Reading Test; HAM-A = Hamilton Anxiety Scale; PAS = Parkinson’s disease Anxiety Scale; HAM-D = Hamilton Depression Scale, SAS = Starkstein Apathy Scale; IADL = Instrumental Activities of Daily Living; M = Male; F = Female; IQCODE = Informant Questionnaire on Cognitive Decline in the Elderly (IQCODE); DKEFS = Delis-Kaplan Executive Function System; ns = Non statistically significant

#### Clinical and cognitive measures

All participants were administered the MoCA for global cognition (Gill et al., 2008); Hamilton Depression Scale (HAM-D) (Hamilton, 1960), Hamilton Anxiety Scale (HAM-A) (Maier et al., 1988) and Parkinson’s Disease Anxiety Scale (PAS) (Leentjens et al., 2014) for depression and anxiety; Starkstein Apathy Scale (SAS) (Starkstein et al., 1992) for apathy; MINI-plus (Sheehan et al., 1998), for psychological symptoms; Cambridge Contextual Reading Test (CCRT) (Beardsall, 1998) for premorbid IQ; and Informant Questionnaire on Cognitive Decline in the Elderly (IQCODE) (Jorm et al., 1991). Participants with PD also completed the Movement Disorder Society-Unified Parkinson’s Disease Rating Scale (MDS-UPDRS) for PD symptom severity (Goetz et al., 2008) and the Lawton Instrumental Activities of Daily Living (IADL) for functional ability (Graf, 2008).

The identification of PD-MCI was determined according to the level 2 Movement Disorder Society (MDS) task force criteria (Litvan et al., 2012). A comprehensive neuropsychological battery of tests in five cognitive domains of attention/working memory, memory, language, executive function and visuospatial function was administered. We included two tests from each of the cognitive domains based on one of the recommended tests within the MDS task force criteria. In brief, the test battery included Stroop Word and Color Test Interference (Golden et al., 1978) and Trail Making Test A and B (Lezak et al., 2012) for attention/working memory, Hopkin’s Verbal Learning Test-Revised (Benedict & Brandt, 2007) and Brief Visual Memory Test-Revised (Benedict & Brandt, 2007) for memory, Delis-Kaplan Executive Function System (D-KEFS) (Delis et al., 2001) for semantic fluency and Boston Naming Test (Roth, 2011) for language, D-KEFS card sorting test, phonemic and semantic switching test for executive function, and Benton’s Judgment of Line Orientation (Benton et al., 1994) and CLOX Clock drawing (Royall et al., 1998) for visuospatial function. PD-MCI was diagnosed when PD participants performed 1.5 *SD* below the normative mean score on at least two neuropsychological tests in one or more cognitive domains out of five cognitive domains recommended to include for testing in PD (Litvan et al., 2012) and having high functional independence based on the IADL scale scoring matrix that ranged from 0 to 8 (Graf, 2008).

### Verbal fluency tasks outside the scanner

The Delis-Kaplan Executive Function System (D-KEFS) verbal fluency assessment involved letter (phonemic) fluency, semantic fluency, and semantic switching. For each trial, participants were asked to generate as many words as they could within 60 s. For letter (phonemic) fluency, participants were asked to generate words starting with a specific letter of the alphabet. Letter cues were F, A, and S. For semantic fluency, participants were asked to generate words within each category (animals and boys’ names). For semantic switching, participants were asked to switch back and forth between saying names of fruits and furniture. The number of correct responses and response time for each condition were measured. A correct response was scored as one, and an incorrect response (e.g., words that did not belong to the target category, grammatical variants, or repeated words) was given a score of zero.

#### Semantic fluency task (fMRI task)

During the in-scanner task, each participant was prompted with different instructions aligned to the following four conditions with a timing of 11.5 s per condition: (1) Rest (“Think of nothing”), (2) Automatic Speech (e.g., “Name the months of the year”), (3) Overt unpaced semantic fluency (e.g., “Name musical instruments”) and (4) Overt unpaced semantic switching (e.g., “Name colors and male first names (switch)”). The categories used for the semantic fluency and semantic switching conditions were selected from category norms (Van Overschelde et al., 2004). The task consisted of two sessions, a total of 64 trials (16 per condition). For each trial, the instructions were first displayed on the screen (4000 ms), and then a green dot appeared on the screen, during which participants were asked to provide their responses loudly and clearly (except during rest) until the dot disappeared (11,500 ms). Participants also completed a practice session that included two trials for each condition that were not listed in the actual task. Responses were recorded through the MR compatible optoactive acoustic microphone (Optoacoutics, www.optoacoustics.com). The task was presented using Cogent 2000 (Welcome Laboratory of Neurobiology) and MATLAB 2018a (The MathWorks Inc., Natick, MA). For behavioral assessments, the voice recordings were extracted per trial and scored using the same methodology that was used for the D-KEFS verbal fluency tasks described above.

#### MRI Acquisition

MRI scans were performed on a Siemens 3T Prisma (Siemens, Erlangen) using a 20-channel head coil. A total of 125 BOLD sensitive gradient echo-planar images (EPIs) were acquired during each block with 3 dummy scans at the start of each session (TE = 30 ms, TR = 2050 ms, flip angle = 80°, axial slices = 50, slice thickness = 2.6 mm, contiguous slices, field of view = 190). Three-dimensional high-resolution structural T1-weighted MP2RAGE scans were also obtained (TE = 2.05 ms, TI = 700 ms, FA = 3, field of view = 256 × 240 × 176 mm^3^).

#### MRI Processing

All fMRI preprocessing and analyses were conducted using SPM12 (Wellcome Trust Centre for Neuroimaging, London, UK) and MATLAB 2018a (The Mathworks Inc., Natick, MA). The first three functional volumes were discarded to allow for steady-state magnetisation. The EPI images were realigned and unwarped (Andersson et al., 2001). The derived six-motion fingerprint parameters using the Motion Fingerprint Toolbox (Wilke, 2012) were used as regressors. Each participant’s EPI and T1 images were coregistered, and then T1 was normalized to a Diffeomorphic Anatomical Registration Through Exponentiated Lie algebra (DARTEL) template created from all participants (Ashburner, 2007). Normalized functional volumes were smoothed with a Gaussian Kernel of 6.5 mm^3^ (FWHM). The gray matter mask based on the structural template was overlaid to focus on the regions within the gray matter.

### Statistical analysis

For all clinical characteristic variables, assessment of normality was performed with the Shapiro–Wilk test. Missing data were handled by using complete case analysis as recommended by Kang et al. (2013). We performed ANOVA/Kruskal–Wallis H tests to compare the differences between PD-MCI, PD-NC and non-PD patients in SPSS v25 (IMBL Armonk, New York, USA). Repeated measures ANOVA was used to analyze the effect of group and condition. A significance level of p < 0.05 was used.

For the fMRI analysis, GLMs were created with realignment parameters using the six motion fingerprint parameters (Wilke, 2012, 2014). Four conditions during the task were created as a contrast at the first level and were compared between the groups. Each trial of 11.5 s was analyzed as a block. Resting and automated conditions were used as a baseline in the first-level design. An uncorrected significance level of *p* < 0.001 and cluster size FWE of *p* < 0.05 in SPM12 were accepted. The association between significant clusters and verbal fluency cognitive measures was examined using correlation analysis. A significance value was adjusted using the Bonferroni correction.

## Results

### Clinical characteristics

Of 37 PD participants, 13 participants (35%) fulfilled the MDS level 2 criteria for PD-MCI (Litvan et al., 2012). The PD-MCI and PD-NC groups did not differ significantly on demographic and clinical characteristics (Table 1) or on PD-specific measures. As expected, participants in the PD-MCI group performed significantly worse on the MoCA (χ^2^ (2) = 17.309, *p* < 0.001) than the PD-NC and non-PD groups, while there was no difference between the PD-NC and non-PD groups. Of 13 PD-MCI patients, 11 demonstrated impairments in multiple cognitive domains. The remaining two participants demonstrated single domain language impairment. The most commonly impaired cognitive domains in PD-MCI were executive function (N = 8), followed by attention and working memory (N = 7), memory (N = 7), language (N = 6), and visuospatial function (N = 2). All males and females scored ≥ 6 in the IADL.

#### Behavioral results

When comparing outcomes from the DKEFS fluency assessments, overall, participants in the PD-MCI group performed significantly worse than those in the PD-NC and non-PD groups (see Table 1). For the tasks performed inside the scanner, the mean number of correct responses for semantic fluency (χ^2^ (2) = 11.53, p = 0.003) and semantic switching (χ^2^ (2) = 14.148, p = 0.001) significantly differed between PD-MCI, PD-NC and non-PD (Table 2). Automatic responses did not differ between the three groups. Post hoc analysis identified a significantly lower mean number of correct responses during semantic fluency in the PD-MCI group than in the PD-NC and non-PD (p < 0.01) groups.

**Table 2:** Average number of responses for each condition per group during fMRI

	PD-MCI (N = 13)	PD-NC (N = 24)	Non-PD (N = 20)	p-value	Post-hoc analysis
Automatic^a^	13.01(3.49)	15.53(5.52)	14.03(4.01)	ns	
Semantic^b^	5.09 (0.73)	6.21(0.92)	6.21(1.25)	0.003	PD-MCI < PD-NC**, PD-MCI < Non-PD**
Semantic Switching^b^	2.40(1.08)	3.49 (1.03)	4.07(1.24)	0.001	PD-MCI < PD-NC*, PD-MCI < Non-PD**

^a^ANOVA (Analysis of Variance) Test; ^b^Kruskal-Wallis H Test

Table of average number of responses (SD) for automatic, semantic, semantic switching during the semantic fluency fMRI task for 15s. Statistically significant results for post-hoc analysis are indicated as * p < 0.05, ** p < 0.01, *** p < 0.001. *Note.* PD-MCI = Parkinson’s disease participants with mild cognitive impairment; PD-NC = Parkinson’s disease participants with no cognitive impairment; Non-PD = control participants without PD

During semantic switching, the PD-MCI group also produced a lower mean number of correct responses compared to the PD-NC (p < 0.05) and non-PD (p = 0.001) groups, which did not differ between the PD-NC and non-PD groups. Last, the PD-MCI group failed to respond to 1.77 ± 1.30 trials out of the 64 trials, which was significantly higher (χ^2^ (2) = 12.68, p = 0.002) than the PD-NC (0.38 ± 0.71) and non-PD (0.85 ± 1.90) groups.

#### Imaging results

**Fig. 1:**
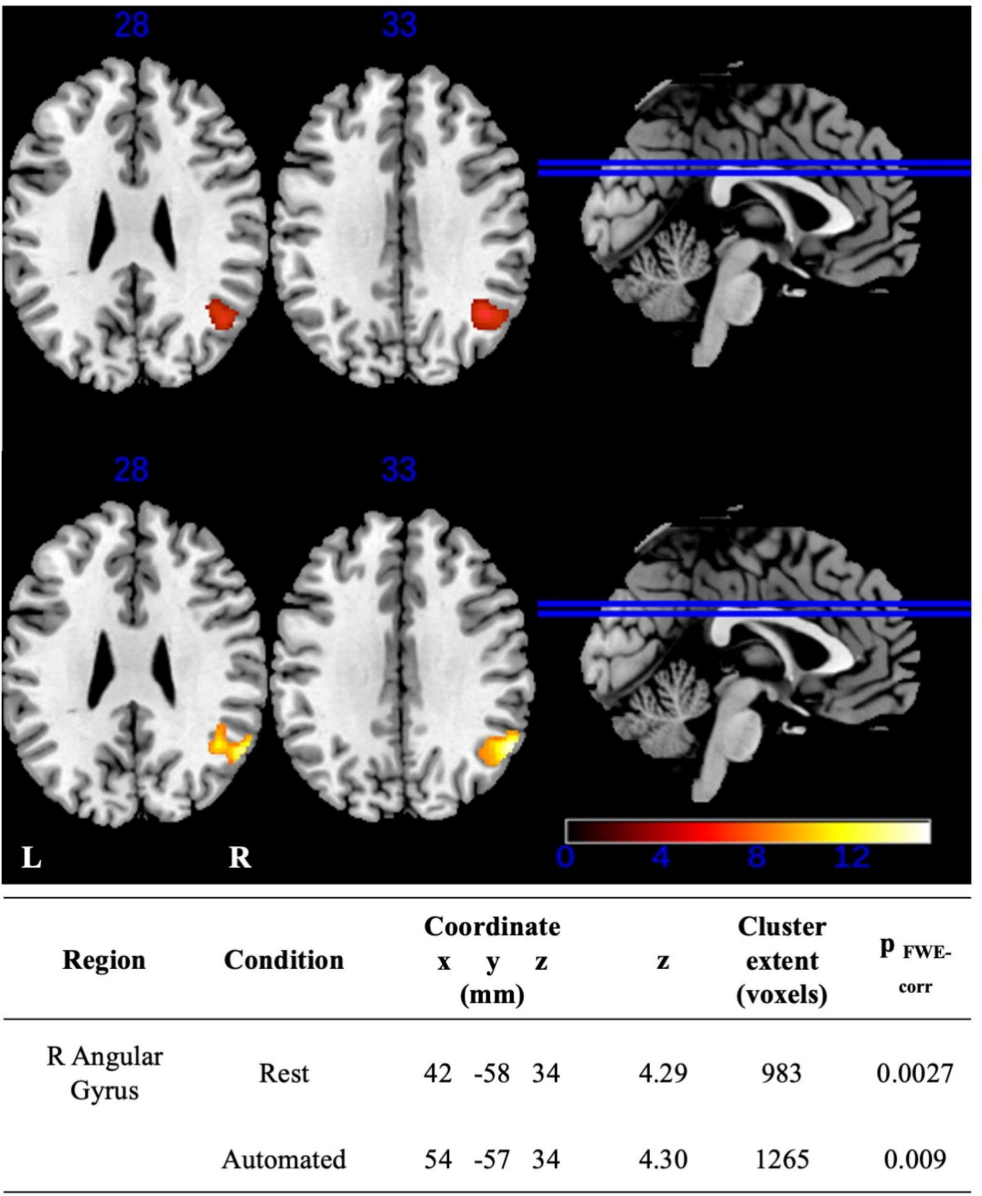
Axial view of main effect of group in category switching greater than rest (top) and category switching compared to automated (bottom) at significance level of p < 0.001 uncorrected, cluster size familywise error (FWE) p < 0.05

**Fig. 2:**
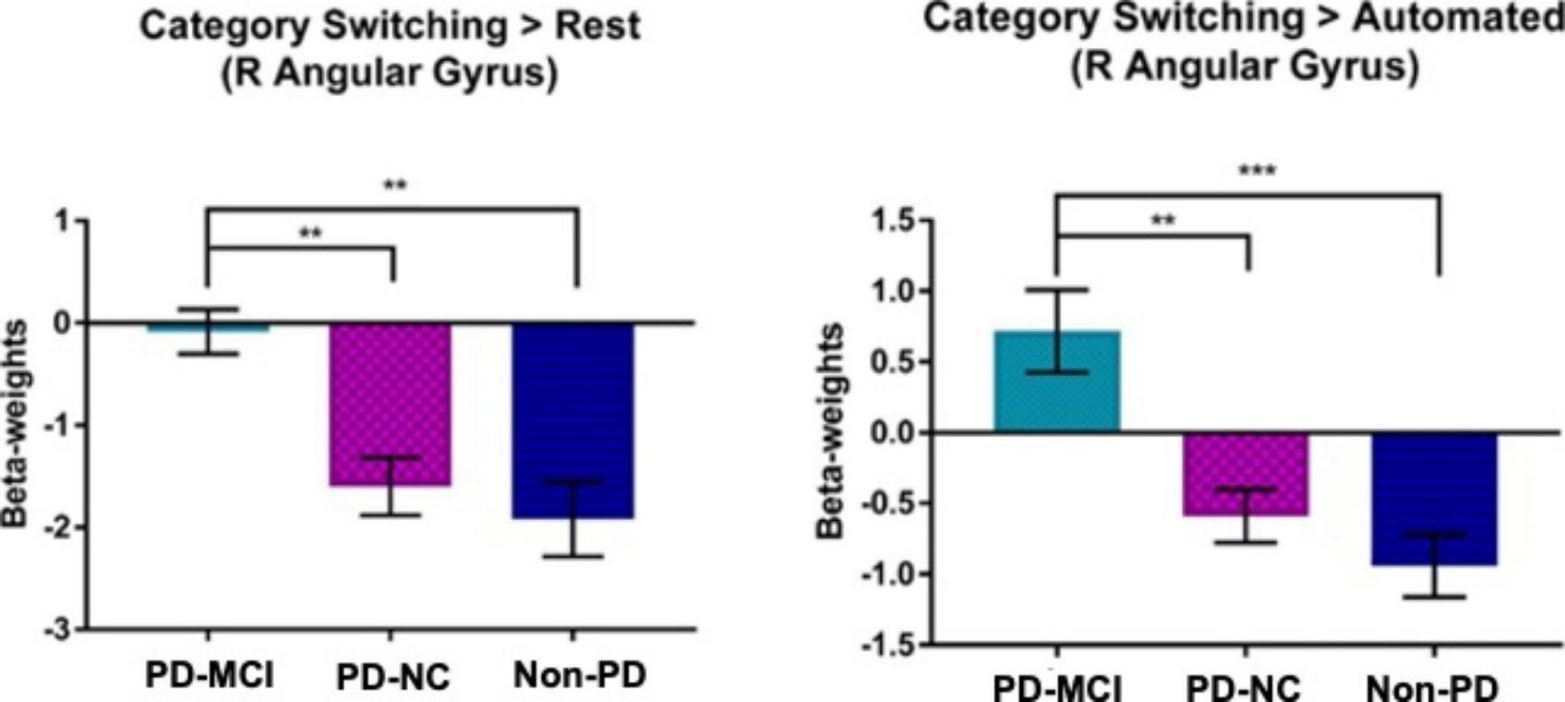
Post-hoc analysis of the category switching > rest (left) and category switching > automated (right). Post-hoc analysis in the right angular gyrus. Bar graph displays mean (SE) beta weighted values. Significant level ** p < 0.01. *Note.* PD-MCI = Parkinson’s disease participants with mild cognitive impairment; PD-NC = Parkinson’s disease participants with no cognitive impairment; non-PD = control participants without PD

The main effect of group on semantic switching compared to the rest condition showed significantly different activation in the right angular gyrus (Fig. 1). Post hoc analysis indicated that right angular gyrus activity was upregulated in the PD-MCI group relative to both the PD-NC group (p < 0.001) and the non-PD group (p < 0.001) (Fig. 1), while no difference was evident between the PD-NC and non-PD groups. There was also a main effect of group for semantic switching compared to the automatic condition in the right angular gyrus (Fig. 1). A follow-up analysis demonstrated that the right angular gyrus was significantly upregulated in the PD-MCI group compared to the PD-NC (p < 0.001) and non-PD (p < 0.001) groups (Fig. 2). There was no significant difference between PD-NC and non-PD.

#### Correlation analysis

**Fig. 3:**
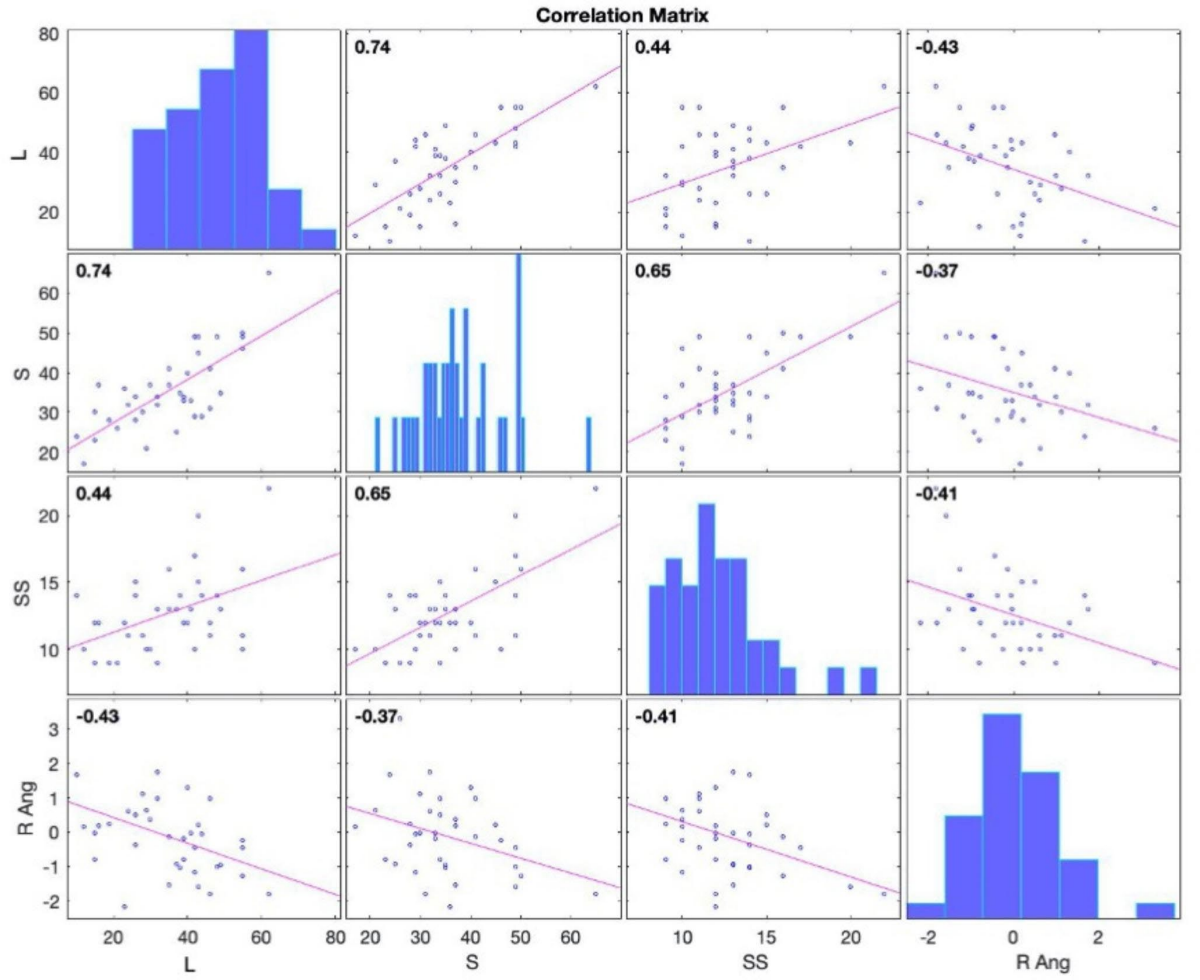
Correlation matrix of scatter plots showing Pearson’s correlation for activation of the right angular gyrus (R Ang) during the category switching > automated and performance of verbal fluency outside the scanner including letter (L), semantic (S) and semantic switching (SS) in participants with Parkinson’s disease (PD)

Correlation analysis was performed between the activity of the right angular gyrus observed during the semantic switching task compared to the automated condition inside the scanner and performance in semantic, semantic switching, and letter fluency tasks conducted outside the scanner in PD participants (Fig. 3). Significant negative correlations were found for all verbal fluency tasks performed outside the scanner: letter (r = -0.43, N = 37, p = 0.001), semantic (r = − 0.37, N = 37, p = 0.02), and semantic switching (r = -0.41, N = 37, p = 0.02).

## Discussion

In this study, we aimed to investigate deficits in verbal fluency in PD-MCI and compare them to PD-NC and age- and sex-matched non-PD participants. Together with behavioral evaluations, this study examined neural mechanisms related to impaired semantic fluency in PD-MCI. At a behavioral level, participants with PD-MCI performed significantly worse than PD-NC and non-PD on semantic and semantic switching fluency, whereas PD-NC performed comparably to non-PD. It is interesting to note that years of education did not differ between the three groups because this minimizes effects due to education. Our findings therefore support previous literature suggesting that verbal fluency deficits are a defining feature of PD-MCI and thus a potential predictor of PDD (Galtier et al., 2017; Williams-Gray et al., 2007). The results also support suggestions that verbal fluency deficits are associated with an increased risk of dementia more broadly (e.g., Sutin et al., (2019)).

PD-MCI participants demonstrated increased activation in the right angular gyrus compared to PD-NC and non-PD groups during the semantic switching task. Interestingly, no differences in brain activation were revealed during the semantic fluency task across groups. This may be because the semantic fluency task is less cognitively demanding than the semantic switching task, which places more substantial demands on executive functions compared to semantic fluency (Baddeley et al., 2001). It is perhaps the taxing combination of executive control, cognitive flexibility, and semantic memory that results in the increased activation of the right angular gyrus and decreased task performance in PD-MCI compared to PD-NC and non-PD. Increased activation was also revealed in the right angular gyrus of PD-MCI participants during semantic switching compared to both the rest and automatic speech conditions, further indicating task-specific hyperactivation in PD-MCI during semantic switching.

When the relationship of the right angular gyrus brain activity to broader fluency performance was considered, there was a significant negative correlation between the activation of the right angular gyrus and verbal fluency tasks, including letter (F, A, S), semantic (e.g., animals, boys’ names) and semantic switching (e.g., fruits and furniture). That is, the greater the number of correct responses produced for each task, the less activity in the right angular gyrus. Therefore, it is apparent that the increased right angular gyrus activity is a predictor of increased verbal fluency deficits identified outside the scanner.

The angular gyrus is located at the junction between the occipital, temporal and parietal lobes and is interconnected bilaterally via the dorsal region of the corpus callosum (Park et al., 2008; Seghier, 2013). It is known to play a significant role in semantic processing, language, complex information integration, memory retrieval, and social cognition (Binder et al., 2009; Seghier, 2013). Semantic processing, in particular, is a function consistently associated with the angular gyrus, with stronger activation typically observed in the left hemisphere (Binder et al., 2009). Apart from its critical role in language, the angular gyrus is also suggested as a cross-modal integrative hub associating spatial awareness and working memory-executive function (Seghier, 2013). However, further research is required to clarify the exact role of the right angular gyrus in verbal fluency to better understand its overall role in semantic memory and executive function.

In the present study, increased activation was observed in the right, but not left, angular gyrus with impaired semantic switching in PD-MCI. While our finding of right as opposed to left angular gyrus activity was unexpected, the involvement of the right angular gyrus in PD-related changes in neural activity is not without precedent. Of note, a recent meta-analysis of whole-brain resting state connectivity in PD found that the most consistent disturbances relative to healthy controls were evident within the posterior inferior parietal lobe (IPL) (Tahmasian et al., 2017). This finding was driven by observations of increased functional connectivity within the bilateral IPL for patients off dopaminergic medication and decreased connectivity for those on medication. The authors suggested that the findings may reflect compensatory mechanisms related to basal ganglia dysfunction associated with the disease. Similarly, Manes et al., (2018) observed increased resting state functional connectivity between the internal globus-pallidus and the angular gyrus bilaterally for PD patients with speech impairments relative to those without speech impairments. The authors attributed these findings to a potential compensatory mechanism and suggested that the findings may reflect increased semantic processing deficits in those with speech impairment given that impaired semantic verbal fluency has previously been shown to correlate with impaired speech (Gago et al., 2009). Similarly, our findings may reflect a compensatory upregulation of activity within the right angular gyrus to compensate for the increasing compromise of cognitive-linguistic function in people with MCI.

Our findings of increased recruitment of a contralateral nontask-dominant region are also supported by previous lesion, aging, and clinical studies (Isaacs et al., 2019; Meinzer et al., 2009; Tinaz et al., 2008) reported increased right inferior/middle frontal activity with worse semantic performance in older adults than in young adults. Isaacs et al., (2019) reported that PD patients showed increased right hemisphere activity during a verbal production task related to verbal fluency compared to non-PD patients. Another study demonstrated an increased recruitment of the right hemisphere in PD patients compared to age-matched and younger healthy controls during the semantic sequencing task (Peran et al., 2009). Compensation for the progressive loss of language function may therefore be reflected by the switch from the left to the right hemisphere, as observed in the present study. This switch may be exacerbated by increased task demands, particularly during the semantic switching task, which requires both semantic memory and executive control. Considering that language function is typically dominated in the left hemisphere, the increased activation for additional recruitment can be related to the inefficient activation of the region in the dominant hemisphere (Braver et al., 2001).

A longitudinal study from the CamPaIGN cohort highlighted that impairment in semantic fluency is a key predictor of PDD, which was assumed to be related to posterior temporal-parietal regions (Williams-Gray et al., 2009). The present study confirmed that increased activity in the parietal region was associated with worse semantic switching fluency in PD-MCI participants. Therefore, our results in PD-MCI indicate that the activation of the right angular gyrus may be a critical indicator for predicting PDD.

### Strengths and Limitations

Stratifying PD-MCI from PD-NC was a great strength of this study, as it allowed for a more in-depth exploration of verbal fluency deficits related to cognitive impairment in PD. For example, Henry & Crawford (2004) found in their meta-analysis that people with PD were impaired on semantic fluency compared to non-PD. However, the results from the present investigation suggest that only PD-MCI participants exhibit impaired semantic fluency compared to non-PD, while verbal fluency in PD-NC is unimpaired. Furthermore, PD-MCI patients showed greater deficits during letter fluency than during semantic fluency, which is contrary to the meta-analysis of Henry & Crawford (2004), which reported the opposite pattern. This finding highlights the importance of looking at PD-MCI when exploring cognition in PD, particularly for the identification of those at risk of dementia. Although our study matched the PD-MCI and PD-NC groups well for potential confounding factors, the relative lack of significance in performance in the PD-MCI group can potentially be a result of a smaller sample size. Given the lack of consistency with the literature, negative results should be interpreted with caution, and further testing in a large sample size is warranted. Another strength was the use of automated speech as a control. The automatic speech condition requires overlearned responses without difficulty; therefore, this condition is used as a baseline for language production and to control for motion artifacts (Birn et al., 2010). Comparing the automatic speech condition to the semantic switching condition enabled us to isolate lexical-semantic processing from speech production.

It is important to consider that the verbal fluency assessments (outside the scanner) used to compare groups were also used to separate groups into PD-MCI and PD-NC. However, PD-MCI is not defined solely by the performance of verbal fluency but rather is defined on comprehensive cognitive testing. PD-MCI participants demonstrated heterogeneous cognitive deficits, and there were also many PD-MCI participants who were not impaired in semantic fluency (N = 9). While we excluded non-PD participants who scored < 19 on the MoCA (cutoff score for dementia), we did not explore whether these non-PD participants experienced MCI. This is because we were not able to administer a full cognitive test battery for non-PD participants. Therefore, a future study may warrant a better understanding of the difference in semantic fluency deficits between MCI patients with and without PD to identify whether the findings are unique to PD. Last, dopaminergic medication can play a considerable role in cognition in PD (Molloy et al., 2006). Another limitation of the present study is therefore that all participants were tested in the ON state, preventing comparison of behavioral and functional results to the OFF state.

## Conclusions

To our knowledge, this is the first fMRI study to investigate neural mechanisms of semantic fluency impairment in PD-MCI. The present study showed that increased functional activity in the right angular gyrus is associated with impaired verbal fluency, particularly in semantic switching, in PD-MCI. This finding supports the view that verbal fluency deficits may be a substantial predictor of cognitive decline related to premorbid PDD. Our results warrant further investigations to understand the progressive cognitive impairment associated with alterations in brain function outlined in this study and its relationship to the development of PDD.

## Electronic supplementary material

Below is the link to the electronic supplementary material.

Supplementary Material 1

Supplementary Material 2

Supplementary Material 3

## Data Availability

The data that support the findings of this study are available from the corresponding author upon reasonable request.

## Code Availability

The data that support the findings of this study are available from the corresponding author upon reasonable request.
