# Supplementary material for: Semantic fluency deficits and associated brain activity in Parkinson’s disease with mild cognitive impairment
Source: Brain Imaging Behav. 2022 Jul 16;16(6):2445–56. doi: 10.1007/s11682-022-00698-7 (PMC9712401; doi:10.1007/s11682-022-00698-7)
Supplement: Supplementary file 1 — Supplementary Material 1 [file 11682_2022_698_MOESM2_ESM.pdf]

Editing Certificate

This document certifies that the manuscript

Semantic fluency deficits and associated brain activity in Parkinson's disease with mild cognitive impairment

prepared by the authors

Jihyun Yang; Katie McMahon; David Copland; Dana Pourzinal; Gerard Byrne; Anthony Angwin; John O'Sullivan; Nadeeka Dissanayaka

was edited for proper English language, grammar, punctuation, spelling, and overall style by one or more of the highly qualified native English speaking editors at AJE.

This certificate was issued on **June 2, 2022** and may be verified on the AJE website using the verification code **50F2-A06B-9627-5568-AC92**.

Neither the research content nor the authors' intentions were altered in any way during the editing process. Documents receiving this certification should be English-ready for publication; however, the author has the ability to accept or reject our suggestions and changes. To verify the final AJE edited version, please visit our verification page at aje.com/certificate. If you have any questions or concerns about this edited document, please contact AJE at support@aje.com.

AJE provides a range of editing, translation, and manuscript services for researchers and publishers around the world. For more information about our company, services, and partner discounts, please visit aje.com.
