# Supplementary material for: Semantic fluency deficits and associated brain activity in Parkinson’s disease with mild cognitive impairment
Source: Brain Imaging Behav. 2022 Jul 16;16(6):2445–56. doi: 10.1007/s11682-022-00698-7 (PMC9712401; doi:10.1007/s11682-022-00698-7)
Supplement: Supplementary file 3 — Supplementary Material 3 [file 11682_2022_698_MOESM4_ESM.docx]

***Author Checklist***

***Brain Imaging and Behavior***

Authors interested in submitting a manuscript to *Brain Imaging and Behavior* may access the individual journal page (<https://www.springer.com/biomed/neuroscience/journal/11682>)

and choose “Submit Online” to access the Editorial Manager website or go directly to <https://www.editorialmanager.com/bior/default.aspx> to login or register.

Each manuscript submitted to *Brain Imaging and Behavior* must be accompanied by a completed checklist. Check only those items applicable to the current submission.

This checklist, along with the accompanying instructional **Author Guidelines**, may be used to help authors write an impactful, scholarly and informative research report. The checklist may also be used by peer-reviewers and/or Editors to ensure that the submitted manuscript meets important journal requirements.

**Style, Format, and References**

✓ The article is formatted in APA style.

✓ The scientific presentation is well-written and free from grammatical or typographical errors.

✓ Abbreviations are defined at first mention and used consistently thereafter.

✓ A word count has been provided and meets the requirements for the specific article type.

**Title Page**

✓ The title is concise and informative.

✓ Author(s) first and last name(s) and affiliation(s) are listed. Middle initials are encouraged.

✓ The corresponding author has provided an email address and telephone number.

**Abstract**

✓ The abstract is between 150 and 250 words and does not contain any abbreviations or citations.

✓ The abstract provides enough information to sufficiently summarize the paper including sample size.

**Keywords**

✓ Topic relevant keywords are listed (3-5).

**Introduction**

✓ Presents a clear rationale for the research being reported.

✓ Provides a focused, up-to-date and scholarly review of the relevant literature.

✓ Links the background literature to the research topic, ensuring a logical flow of ideas.

✓ Provides essential background and contextual information necessary for understanding the main aspects of the study and its significance.

**Methods**

✓ The Study Design is the first subsection of the Methods section and describes how the study was conducted, including all elements of how the data were collected, such as selection criteria and targeted clinical characteristics. Baseline demographics may be under the Results section or in this section, whichever provides greater clarity for readers.

✓ The Statistical Analysis follows the Study Design and clearly describes the statistical procedures used to analyze the data.

✓ Any approach used to correct for multiple tests or comparisons is clearly described and referenced including, for example, FDR, FWE or post-hoc analyses such as Bonferroni or Tukey’s tests.

✓ Data exclusion criteria are provided.

**Neuroimaging**

✓ Vendor information for neuroimaging tools (e.g., MRI, PET, or CT), such as hardware types and sequences names, are reported.

✓ All software used for acquisition, preprocessing and analysis of imaging, behavioral, biomarker, genetic or other information are clearly specified.

✓ Imaging parameters, such as acquisition resolution and coverage/field-of-view, as well as any contrast determining criterion, are reported.

✓ The source and names of any open source software tools are reported, along with key commands, scripts, and their options.

❏ If in-house computing scripts have been used, the programming language, key functions and availability are described in the Methods section.

**Results**

✓ The results are presented objectively and provide a logical and ordered summary of the statistical analyses, without interpretation.

**Tables**

✓ Each table is typed on a separate sheet.

✓ Tables are numbered consecutively using Arabic numbers as referred to in the text.

✓ Footnotes are placed immediately below the table and include a key to any annotations.

**Figures**

✓ Figures sufficiently illustrate the statistical analyses.

✓ Each figure is of a high quality and sufficient in size and clarity to be reproduced.

✓ Each figure is cited in the text and numbered consecutively.

**Figure legends**

✓ Each figure legend is listed on a separate page and numbered consecutively using Arabic numbers (i.e., Fig 1), as referred to in the text.

✓ Figure legends clearly specify all information needed for valid interpretation, including the information represented by the error bars (i.e., SD, SE, CI).

**Halftone Artwork**

❏ Photographs and drawings are of high quality, with a minimum resolution of 300dpi.

❏ Combination halftone and line art have a minimum resolution of 600 dpi.

❏ Any magnification used in the photographs are indicated by a scale bar within the figure.

**Discussion**

✓ The discussion interprets the significance of the findings without reiterating the results.

✓ Limitations in study design or with statistical analyses are discussed.

✓ New knowledge, insights or understanding are provided.

**Conclusions**

✓ The major findings of the manuscript are briefly restated.

**Acknowledgements**

✓ People are acknowledged who have provided a significant contribution to the research report but do not meet the requirements for authorship.

**Funding Sources**

✓ All funding sources directly or indirectly supporting the research have been acknowledged, including grant numbers where appropriate.

**Conflict of Interest and Disclosure**

✓ Potential conflicts of interest, financial or otherwise, have been disclosed.

✓ The submitted work has not been published previously and is not being considered for publication elsewhere.

✓ Material has not been reproduced from prior publications, whether by the same or different authors. Any previously published material is explicitly quoted and referenced.

✓ Prior publications or other submissions on the same data set or problem have been disclosed.

✓ Each author has read and completed all sections of the Author Disclosure Form.

**Human or Animal Subjects**

❏ I verify that this research did not involve the use of human or animal subjects.

OR

❏ I verify that appropriate Institutional Review Board (IRB), Institutional Animal Care and Use Committee (IACUC), and/or ethics committee approval has been obtained for the use of human or animal subjects.

✓ I verify that written informed consent was obtained from all research particpants.
